# Proposal of a Risk-Stratification Platform to Address Distinct Clinical Features of Pediatric Kaposi Sarcoma in Lilongwe, Malawi

**DOI:** 10.1200/JGO.17.00054

**Published:** 2017-12-22

**Authors:** Nader Kim El-Mallawany, William Kamiyango, Jimmy Villiera, Jeremy S. Slone, Carrie L. Kovarik, Liane R. Campbell, Anurag K. Agrawal, Dirk P. Dittmer, Anthony B. Eason, Saeed Ahmed, Gordon E. Schutze, Michael E. Scheurer, Peter N. Kazembe, Parth S. Mehta

**Affiliations:** **Nader Kim El-Mallawany**, **Jeremy S. Slone**, **Michael E. Scheurer**, **Saeed Ahmed**, **Gordon E. Schutze**, and **Parth S. Mehta**, Baylor College of Medicine; **Nader Kim El-Mallawany**, **Jeremy S. Slone**, **Michael E. Scheurer**, and **Parth S. Mehta**, Texas Children’s Cancer and Hematology Centers, Houston, TX; **William Kamiyango**, **Jimmy Villiera**, **Saeed Ahmed**, and **Peter N. Kazembe**, Baylor College of Medicine Children’s Foundation Malawi; **William Kamiyango**, **Jimmy Villiera**, and **Peter N. Kazembe**, Kamuzu Central Hospital, Lilongwe, Malawi; **Carrie L. Kovarik**, University of Pennsylvania, Philadelphia, PA; **Liane R. Campbell**, Baylor College of Medicine Children’s Foundation Tanzania, Baylor International Pediatric AIDS Initiative at Texas Children’s Hospital, Mbeya, Tanzania; **Anurag K. Agrawal**, Children’s Hospital and Research Center Oakland, Oakland, CA; and **Dirk P. Dittmer** and **Anthony B. Eason**, Lineberger Comprehensive Cancer Center, University of North Carolina, Chapel Hill, NC.

Kaposi sarcoma (KS) is among the three most common childhood malignancies in regions of Africa where human herpesvirus-8 (HHV-8)/KS-associated herpesvirus are endemic.^1-5^ In contrast to adult disease, pediatric KS staging classifications, risk-stratification systems, and treatment paradigms remain poorly defined.^6-12^

Prior studies suggest that the clinical features associated with survival in childhood KS are distinct from those in adults.^6-14^ In our retrospective study of factors associated with event-free survival (EFS) and overall survival (OS) in pediatric KS, multivariable analysis demonstrated that visceral disease and disseminated skin/oral presentation (defined as ≥ 20 hyperpigmented lesions in a widespread distribution) were independent risk factors for death and inability to achieve EFS with a minimally myelosuppressive regimen that contains bleomycin and vincristine (BV), which is commonly available even in low-income settings.^12^ Patients with woody edema had low EFS but did not experience increased mortality.^12^ Lymphadenopathic involvement in children was associated with the highest rates of long-term complete remission (CR).^12^

Few pediatric KS occurrences develop outside of sub-Saharan Africa; thus, the AIDS Clinical Trial Group (ACTG) tumor extent (T), immune status (I), and systemic illness (S) staging classification (ie, TIS), which was based on HIV-related KS occurrences in adults in the United States, has not shown consistent prognostic significance for pediatric KS in Africa.^7,8,10,12-14^ Therefore, we propose a pediatric-specific KS staging classification to potentially serve as a working risk-stratification platform for children with KS in sub-Saharan Africa.

## Proposed Pediatric-Specific KS Staging Classification

The Lilongwe pediatric KS staging classification was devised from an analysis of clinical covariables associated with EFS and OS from our previously published retrospective observational cohort of HIV-infected children and adolescents (younger than 18 years) with histologically or clinically diagnosed KS.^12^ Similar to stage-stratified approaches for adult KS in high-income countries, this staging system is based solely upon the extent of KS disease and not on immunologic or systemic factors.^15^

We define four distinct groups of patients. Stage 1 is labeled Mild KS, with disease limited to skin, flat oral mucosa lesions, and/or flesh colored subcutaneous nodules, with fewer than 10 lesions total. Stage 2 is labeled Lymphadenopathic KS, including patients with lymph node involvement, nodular oral lesions, facial edema, conjunctival lesions, exophytic mass, or 10 to 19 hyperpigmented skin/oral lesions (or any patient who does not meet criteria for stages 1, 3, or 4). Stage 3 is labeled Woody Edema KS, defined as woody edema with or without any of the characteristics of stages 1 or 2; this stage is subdivided into two parts: 3A, edema that involves less than 10% of estimated body surface area (BSA), and 3B, edema that involves more than 10% of estimated BSA (estimates were made with the Wallace Rule of 9s, which is used in burn victims). Stage 4 is labeled Visceral and/or Disseminated Skin/Oral KS, defined as clinical pulmonary or abdominal visceral involvement and/or ≥ 20 hyperpigmented skin/oral lesions in a widespread distribution (excluding flesh-colored subcutaneous nodules), with or without any of the criteria of the other stages.

The following are important footnotes to the proposed staging classification: (1) Coalescing or confluent hyperpigmented skin lesions localized to an anatomic region count as one lesion per cluster. (2) Detailed working definitions for visceral KS in the absence of bronchoscopy and endoscopy have been described previously.^12^ (3) Although the majority of patients with stage 2 disease had lymphadenopathy, this stage does not require lymph node involvement. (4) Because presentation with moderate-severe cytopenias was not associated with inferior survival, cytopenias were not incorporated into the classification.^12^

ACTG TIS staging criteria definitions were evaluated for comparison on the basis of published guidelines.^16^ Induction failure in our cohort was defined as the inability to achieve more than 90% reduction in size (subjectively determined) of all lesions after induction-phase treatment with BV (four cycles). Treatment regimens, supportive care, and statistical analyses have been described previously.^12^ Ethics committee approvals were obtained in Malawi and by the collaborating institution in the United States.

Sixty-eight patients had their diseases fully staged by using these criteria. No patients had stage 1 disease; 37 (54%) had stage 2; 14 (21%) had stage 3; and 17 (25%) had stage 4 disease. Clinical characteristics and treatment response differed distinctly by stage (Table 1). Statistically significant characteristics specific to stage 2 included young age, lymph node involvement, and low rates of induction failure. Those classified with stage 3 disease were significantly older and less likely to have lymph node involvement. Patients with stage 4 disease demonstrated the lowest mean CD4 count and an association with oral mucosal involvement. We observed that 19 (51%) of 37 patients with stage 2 disease were already on combination antiretroviral therapy (cART) at the time of KS diagnosis. Of those 18 patients who were not on cART, 13 (72%) presented with moderate-to-severe cytopenias—a clinical presentation in which the disease responds favorably to chemotherapy but can progress rapidly if chemotherapy is not initiated promptly.

**Table 1:**
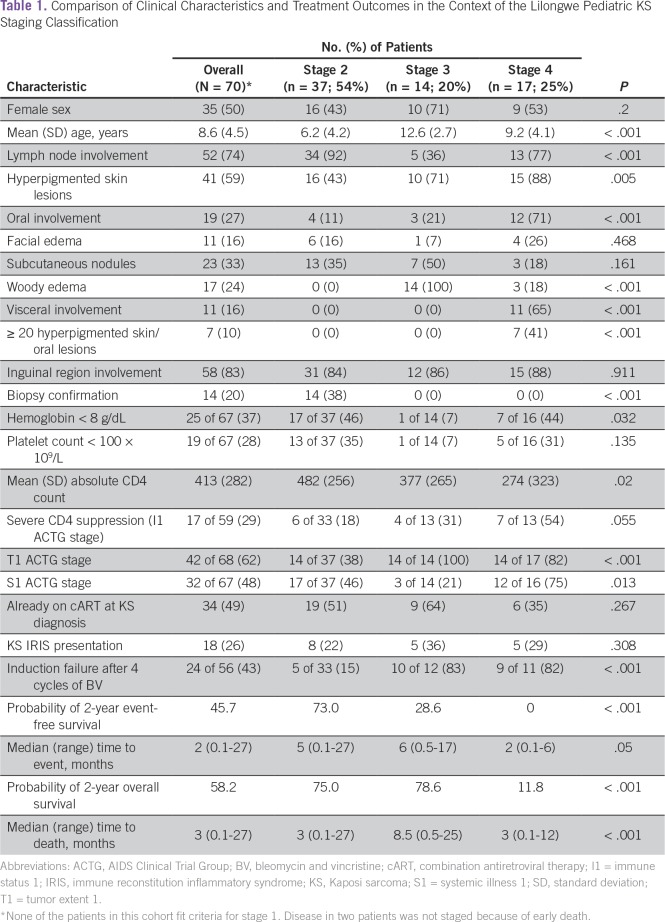
Comparison of Clinical Characteristics and Treatment Outcomes in the Context of the Lilongwe Pediatric KS Staging Classification

When applied to this cohort, the pediatric-specific staging classification determined significantly different outcomes for both EFS and OS (Appendix Fig A1A, online only). With a median follow-up time of 28 months (range, 15 to 50 months), patients who had stage 2 KS demonstrated a probability of 2-year EFS and OS of 73% and 75%, respectively, compared with patients who had stage 3 KS (EFS and OS of 28.6% and 78.6%, respectively) or stage 4 KS (EFS and OS of 0% and 11.8%, respectively; *P* < .001). The two who survived with stage 4 disease were treated with intensified chemotherapy regimens that included doxorubicin plus BV (ABV), and they remained alive on lifelong cART at 17 and 27 months, respectively, from their dates of KS diagnoses.

Causes of death varied by clinical stage. Of the 10 patients with stage 2 disease who died, KS was the cause in four, whereas the remaining six died as a result of complications of uncontrolled HIV (n = 4 late deaths in patients in CR, n = 2 early deaths from concurrent opportunistic infection). Among the 15 deaths in patients with stage 4 disease, all were attributed to refractory/progressive KS. Three patients received ABV (after disease failed to respond to BV) and survived 4, 9, and 12 months, respectively. The remaining 12 patients received treatment before the availability of doxorubicin; the median time to death in this group was 3 months (longest survival, 7 months). All four deaths in patients with stage 3 disease were a result of non-KS causes.

A comparison of the Lilongwe pediatric KS staging classification with the ACTG T staging classification is depicted in Appendix Figs A1A and A1B. Although analysis of survival to compare T1 versus T0 stages demonstrated a statistically significant difference in EFS, the difference in OS was not significant.

## Formulating a Risk-Stratification Platform to Guide Therapy

Because of the clinical heterogeneity that is inherent to all childhood malignancies, risk stratification is an essential element that guides the formation of treatment paradigms in pediatric oncology worldwide. Distinct heterogeneity exists in pediatric KS as well, which provides the impetus for the formation of pediatric-specific systems.^12^ In this retrospective cohort, the proposed staging classification differentiated clinical patterns of disease associated with contrasting survival outcomes. Thus, the classification presents a potential platform for the development of risk-stratified treatment protocols in sub-Saharan Africa.

The relevance of this risk-stratification system stems from prognostication of which patients can achieve favorable survival with the minimally myelosuppressive BV chemotherapy regimen versus which patients will require a more intensive chemotherapeutic strategy. Given the limited capacity to deliver optimal supportive care for patients with cancer throughout Africa, it is critical to identify patients— especially patients with underlying immune suppression from HIV—whose disease can be treated successfully with less myelosuppressive regimens.

The pediatric-specific staging classification highlights the most common clinical feature of childhood KS in central and eastern Africa: lymph node involvement.^7-10,12^ The clinical presentation of patients classified with stage 2 disease is characterized by a high rate of KS lymphadenopathy, a clinical presentation that can be difficult to identify in the absence of prototypical skin, oral, or edematous KS lesions. Heightened awareness of lymphadenopathic KS is important, because these children often present as critically ill with severe cytopenias. Given that the reconstitution of cellular immunity may require several months with cART, we feel that the timely initiation of BV chemotherapy in addition to cART is critical in this group and has a high potential to achieve a durable CR.

Compared with the ACTG staging system used worldwide for HIV-related KS in adults, the proposed pediatric-specific system divides the T1 population into those with woody edema (stage 3, low EFS/relatively high OS) and those with visceral disease (stage 4, low EFS/low OS). In addition, children with disseminated skin disease, whose disease would be considered T0 in the ACTG system because of the absence of visceral involvement, had disease defined as stage 4 in the proposed pediatric classification because of the ≥ 20 hyperpigmented skin/oral lesions. These patients experienced extremely high mortality rates, contrary to descriptions of adult KS in whom the T0 presentation of more than 50 skin lesions is not uncommon and is not necessarily associated with poor survival.^12,15-17^

Woody edema is the defining characteristic of patients classified with stage 3 disease; it tended to occur in older children and adolescents (mean age, 12.6 years), which resembled a common clinical presentation of adults with KS in Malawi.^17^ Stage 3 disease is characterized by an indolent course, so the combination of chemotherapy and cART more often achieved stable disease control, rather than CR, and improved quality of life. Although a minority (n = 4) of patients with stage 3 disease achieved CR with BV (EFS, 28.6%), it is notable that all four patients presented with less than 10% of their BSA affected by edema, which most often was localized to the anterior aspect of the inner thigh. Thus, studies that involve larger cohorts may consider subdivision of stage 3 into A and B substages on the basis of the extent of estimated BSA involved. In our experience with extensive woody edema, BV seemed to provide similar partial remission/stable disease status compared with more intensive regimens (ABV or paclitaxel), although small patient numbers preclude definitive analysis. Ultimately, the most reasonable goal may be to optimize quality of life with the least intensive chemotherapy possible.

Stage 4 disease was typified by an 88% mortality rate; all deaths were attributed to KS. Severe CD4 suppression was common in this group, but it was not a defining characteristic. In our experience, patients rarely survived more than 6 months unless the chemotherapy regimen was intensified to include doxorubicin. With time, as the clinical pattern of stage 4 disease became apparent to our team, earlier intensification to ABV was instituted and benefited two patients, who remain alive and in CR. However, it is difficult to predict whether the intensification of chemotherapy to ABV or paclitaxel will consistently achieve long-term disease control. A limitation lies in the challenge of prospective diagnosis of visceral disease in the absence of endoscopy and bronchoscopy in low-income settings.

Mild KS with fewer than 10 skin/oral/subcutaneous lesions was designated stage 1 in our classification. No patient in the cohort fit this category, and the explanation for the rarity of this phenomenon is uncertain. There exists bias in a study performed at a tertiary care facility, where access to care is limited to referral patterns and outreach networks. Potentially, HIV-infected children with mild KS do not get referred or identified if cART alone (prescribed by a practitioner at the local health care facility) induces remission. Alternatively, we hypothesize that mild KS is uncommon in children in regions where HHV-8 is endemic. Nonetheless, we feel that it is important to include this group as a possible clinical scenario, because these patients theoretically could be spared chemotherapy and treated with cART alone.

The biggest limitation to this proposed system lies in the use of the same retrospective cohort to derive and evaluate the staging classification. A prospective, multicenter, and preferably international study is required to validate the proposed staging classification. However, because of the void of established treatment paradigms and the urgent clinical need in the face of continued high numbers of pediatric patients with KS, we feel that it is important to preliminarily establish working staging and risk-stratification systems.^4^ We also recognize that, because KS can involve virtually any organ, the proposed clinical staging paradigm is not exhaustive and may evolve.

The proposed Lilongwe pediatric KS staging classification differentiates clinical patterns of disease that may provide a platform for risk stratification and may guide therapeutic strategies. It identifies groups of patients (ie, those with stages 2 and 3 disease) who can achieve favorable survival with the minimally myelosuppressive BV chemotherapeutic regimen as well as those (ie, patients with stage 4 disease) who require alternative (and likely intensified) regimens to potentially improve survival outcomes. In addition, the classification establishes a potential, albeit uncommon, subgroup of patients who theoretically could be treated with cART alone (without chemotherapy). Prospective validation of this staging classification is the next step to define optimal treatment paradigms and improve OS outcomes for children and adolescents with KS.

## Figures and Tables

**Appendix:**
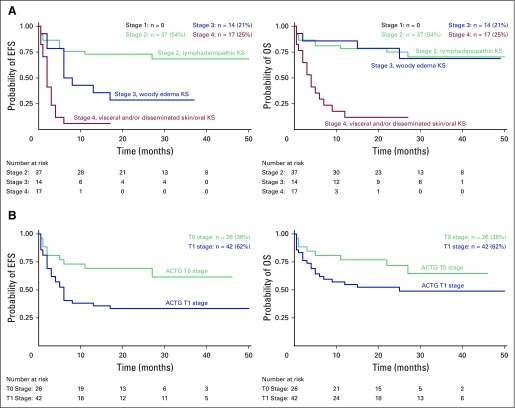
Kaplan-Meier survival curves for children and adolescents with Kaposi sSarcoma (KS) stratified by stage. The probability of 2-year event-free survival (left panels) and overall survival (right panels) stratified by (A) the Lilongwe Pediatric KS Staging Classification (EFS and OS, *P* < .001 for each) (1A) as well as based uponor (B) the AIDS Clinical Trial Group (ACTG) T1 versus T0 tumor staging (1B). (T0 and T1 comparison: EFS *P* = .0138 and OS *P* = .1155).
